# Anti-nausea effects and pharmacokinetics of ondansetron, maropitant and metoclopramide in a low-dose cisplatin model of nausea and vomiting in the dog: a blinded crossover study

**DOI:** 10.1186/s12917-017-1156-7

**Published:** 2017-08-16

**Authors:** Hannah Kenward, Jonathan Elliott, Terry Lee, Ludovic Pelligand

**Affiliations:** 1 Department of Comparative Biomedical Sciences, Royal Veterinary College, Hawkshead Lane, North Mymms, Hatfield, Herts AL9 7TA UK; 2 Department of Clinical Sciences and Services, Royal Veterinary College, Hawkshead Lane, North Mymms, Hatfield, Herts AL9 7TA UK; 3 Analytical Services International (ASI), St Georges, University of London, Cranmer Terrace, London, SW17 0RE UK

**Keywords:** Nausea, Emesis, Cisplatin, Antiemetic, Maropitant, Ondansetron, Metoclopramide, Biomarker, Arginine vasopressin, Cortisol

## Abstract

**Background:**

Nausea is a subjective sensation which is difficult to measure in non-verbal species. The aims of this study were to determine the efficacy of three classes of antiemetic drugs in a novel low dose cisplatin model of nausea and vomiting and measure change in potential nausea biomarkers arginine vasopressin (AVP) and cortisol. A four period cross-over blinded study was conducted in eight healthy beagle dogs of both genders. Dogs were administered 18 mg/m^2^ cisplatin intravenously, followed 45 min later by a 15 min infusion of either placebo (saline) or antiemetic treatment with ondansetron (0.5 mg/kg; 5-HT_3_ antagonist), maropitant (1 mg/kg; NK_1_ antagonist) or metoclopramide (0.5 mg/kg; D_2_ antagonist). The number of vomits and nausea associated behaviours, scored on a visual analogue scale, were recorded every 15 min for 8 h following cisplatin administration. Plasma samples were collected to measure AVP, cortisol and antiemetic drug concentrations.

**Results:**

The placebo treated group vomited an average number of 7 times (range 2–13). None of the dogs in either the ondansetron or maropitant treated groups vomited during the observation period. The onset of nausea-like behaviour in the placebo-treated group occurred at t_3.5h_ and peaked at t_4.75h_ with nausea behaviour score of 58.5 ± 4.6 mm. Ondansetron and maropitant reduced overall the area under the curve of nausea behaviour score by 90% and 25%, respectively. Metoclopramide had no effect on either vomiting or nausea.

Cisplatin-induced nausea and vomiting caused concomitant increases in AVP and cortisol. In the placebo-treated group, AVP and cortisol increased from t_2.5h_, peaked at t_5h_ (11.3 ± 2.9 pmol L^−1^ and 334.0 ± 46.7 nmol/L, respectively) and returned to baseline by t_8h_. AVP and cortisol increases were completely prevented by ondansetron and only partially by maropitant, while metoclopramide had no effect. The terminal half-lives (harmonic mean ± pseudo SD) for ondansetron, maropitant and metoclopramide were 1.21 ± 0.51, 5.62 ± 0.77 and 0.87 ± 0.17 h respectively.

**Conclusions:**

5-HT_3_ receptor antagonist ondansetron demonstrates the greatest anti-emetic and anti-nausea efficacy of the three drugs. AVP and cortisol appear to be selective biomarkers of nausea rather than emesis, providing a means of objectively measuring of nausea in the dog.

## Background

Nausea is a subjective sensation induced by a variety of emetic stimuli and usually preceeds emesis. Nausea is a graded response with a dynamic threshold influenced by a variety of factors [1], unlike emesis, which is an all or nothing event occurring when emetic stimuli surpass the threshold required to activate the emetic reflex. Defining nausea in animals is inherently problematic due to its subjective nature and the inability of an animal to verbalise the sensation that they experience. Whether or not animals experience the sensation of nausea in the same way as people do is a contentious issue [2]. However, for the purposes of this article the term nausea will be used to denote the aversive state and prodromal response induced by the administration of a known emetogenic substance. Nausea-like behaviour refers to observable behaviours in animals that occur during the aversive state.

Antiemetic drugs work by blocking the emetic pathway at various points preventing the emetic reflex. Three major classes of anti-emetics are currently available for the treatment of chemotherapy-induced nausea and vomiting: 5-hydroxytryptamine_3_ (5-HT_3_), neurokinin_1_ (NK_1_) and dopamine_2_ (D_2_) receptor antagonists. All classes of antiemetic drugs have some anti-nausea efficacy [3–5] but are generally less efficacious for the treatment of nausea [6]. Human patients now report that nausea rather than emesis is the most distressing side effect of cancer chemotherapy [7]. The sensation of nausea stems from complex mechanisms involving cortical structures in the forebrain responsible for conscious perception, these mechanisms are thus far poorly characterised [8–10]. This has hindered the development of anti-nausea drugs.

Cisplatin is a platinum based cytotoxic drug used for the treatment of cancer [11]. It is highly emetogenic [12] and has been widely used experimentally to induce nausea and emesis in a variety of species including *Suncus murinus* [13], ferret [14] and the dog [15]. The low dose cisplatin model of nausea and emesis in the dog [16] induces significantly less emesis than previous models which utilise clinical doses of cisplatin [15]. The reduced emesis in the low dose model facilitates recognition of the behavioral signs of nausea and grading of its severity without the bias of frequent emetic events. Therefore, it is a good model for the assessment of the anti-nausea effects of currently available anti-emetic drugs and to relate the concentration of nausea biomarkers to the observed signs of nausea behaviour.

Potential nausea biomarkers, arginine vasopressin (AVP) and cortisol, have been identified as correlates of nausea behaviour in the dog [16]. Increased AVP has been positively correlated to self-reported nausea scores by volunteers, in which, motion sickness is induced [17, 18]. To date, these biomarkers have not been used in experimental intervention studies to assist in objective assessment of nausea.

The principal aim of the current study was to determine the relative efficacy of the representatives of three classes of antiemetic (ondansetron, maropitant, metoclopramide) in the prevention of nausea and emesis in the low dose cisplatin model.

## Methods

### Animals

Animal procedures undertaken were approved by the UK Home Office Animals (Scientific Procedures) Act 1986 (ASPA), Project license 70/7269 with ethical approval of the Royal Veterinary College (RVC) Ethics and Welfare Committee. Eight healthy neutered Beagle dogs (Marshall Farms, North Rose, NY, USA), four male and four females weighing from 6.5 to 11.5 kg, aged 2.5 years old at the start of the studies were used. Dogs were group-housed according to sex on a 12 h light/dark cycle and fed canine Lab diet 5007 (IPS Ltd., London, UK) once daily (100–200 g adjusted as needed to maintain ideal weight). Water was available ad libitum. The dogs were discharged from ASPA and re-homed as pets at the end of the study with the approval of the RVC’s Named Veterinary Surgeon.

### Experimental design

Dogs received cisplatin and antiemetic or placebo treatment in each of the 4 periods of the study. The 4 study periods were blocked by sex and by day with at least a 28 day wash out period being observed between doses of cisplatin. Antiemetic or placebo treatment was randomly allocated during period 1 and a Latin square design was used to determine treatment allocation in the remaining periods so that all dogs received each treatment over the course of the study.

### Jugular vein catheter placement

One day prior to cisplatin administration, a double lumen jugular catheter was implanted under general anesthesia using the methods described in Kenward et al. [16].

### Operator safety

Cisplatin was dispensed with the medical closed system (BD Phaseal, Oxford, UK) under a cytotoxic hood. Appropriate personal protective equipment (PPE) was worn by users when handling cisplatin and contaminated waste. Dogs were quarantined for 10 days following cisplatin treatment, PPE was worn to handle dogs and all waste was considered to be contaminated with cytotoxic material.

### Fluid treatment

**Fig. 1:**
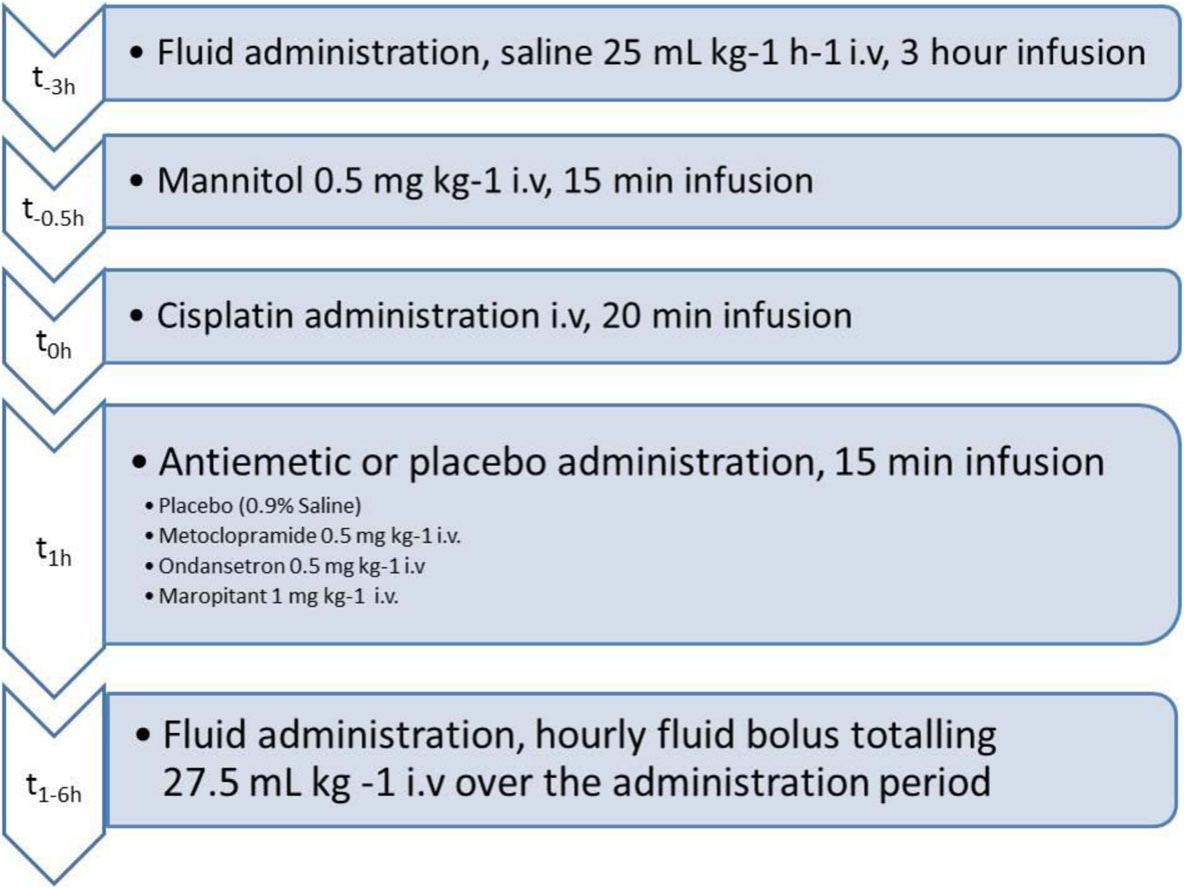
Fluid and drug administration protocol

All dogs were given 0.9% saline and mannitol infusions prior to the administration of cisplatin and an hourly saline bolus post cisplatin through the first lumen of the jugular catheter as described in Kenward et al. [16] to reduce the nephrotoxic effects of cisplatin (Fig. 1).

### Cisplatin administration

The time of initiation of cisplatin infusion was measured in hours and defined as t_0h_ (Fig. 1). At t_0h_, the dogs received 18 mg/m^2^ of cisplatin (Hospira, Leamington Spa, UK). Dogs were weighed on the day preceding the injection and body surface area (m^2^) calculated using the formula:

$$ Dose\ \left({m}^2\right)=\frac{k.{W}^{\raisebox{1ex}{$2$}\!\left/ \!\raisebox{-1ex}{$3$}\right.}}{100} $$

where constant k = 10.1 and W is the weight of the dog in kilograms [19].

The appropriate volume of stock cisplatin solution (1 mg/mL) for each dog was diluted in 0.9% saline to produce a standard volume of 40 mL. The diluted cisplatin solution was administered through the second lumen of the jugular catheter using automatic dispensing syringe at a flow rate of 2.0 mL/min.

### Antiemetic administration

Anti-emetic or placebo administration was completed at t_1h_ following cisplatin treatment. Depending on treatment allocation from the experimental design, dogs received one of the following: 0.5 mg/kg ondansetron (Zofran®, GSK, Brentford, UK), 1 mg/kg maropitant (Cerenia®, Zoetis, Florham Park, NJ, USA), 0.5 mg/kg metoclopramide (Emeprid®, Ceva Santé Animale, Amersham, UK) or 0.9% saline as placebo. All treatments were made up to a standard volume of 15 mL using 0.9% saline and were administered intravenously through the second lumen of the jugular catheter at a flow rate of 1.0 mL/min.

### Behavioural assessment

Prior to the commencement of studies an acclimatization period of 1 month was allowed for the dogs to become familiar with the experimental context. During this period, the observer became accustomed to each individual dog’s normal behaviour, which was used as a baseline reference for behavioural observations. During the 28-day washout interval between the study periods, the dogs were placed in the experimental context weekly to prevent the development of a conditioned response.

Observations of behaviours suggestive of a nausea-like state and the number of vomits were recorded for 7 h following cisplatin treatment. Nausea-like behaviour was recorded by a single trained observer, who was blinded to the treatment the dogs had received. A composite ‘nausea’ score was recorded using a visual analogue scale (VAS) [15]. The observer made a judgment on the severity of the dog’s nausea-like behaviours based on the presence and frequency of one or more of the following during a 15 min time bin; salivation, lip licking, lethargy, restlessness or turning/circling behaviour signaling that vomiting is imminent. A score of 0 mm indicated ‘No ‘Nausea” and a score of 100 mm indicated ‘Worst possible ‘Nausea”. This method of behavioural assessment in dogs was based on a ‘nausea’ visual analogue scale described by de la Puente Redondo et al. [15] and was validated ‘in-house’, for a cisplatin-induced nausea and vomiting, by the authors in Kenward et al. [16].

### Blood sampling

Venous blood samples were collected from the jugular catheter for biomarker analysis at *t* = −3 (baseline), 0, 2.5, 4, 4.5, 5, 5.5, 6, 7, 8 and 24 h following cisplatin administration. Additional samples were collected for pharmacokinetic analysis of the antiemetic drugs at *t* = 1.25, 1.5, 1.75, 2.25, 2.75, 3.25, 4.25, 6.25, 8.25 and 24.25 h following cisplatin administration. Approximately 5 mL of blood was collected for biomarker analysis and 0.5 mL for PK analysis at each time point, giving a maximum of 60 mL collected per dog. Blood was collected into EDTA coated tubes or Lithium Heparin coated tubes as appropriate for subsequent assays. In agreement with ASPA regulations, a maximum of 15% blood volume was withdrawn in any 28 day period, with no more the 10% withdrawn in any 24 h period. Blood sample tubes were placed in an ice bath for a maximum of 10 min before centrifugation at 4000 x g for 15 min. Plasma samples were aliquoted into individual tubes, A protease inhibitor cocktail (15 μM; Sigma Aldrich, UK) was added to tubes containing plasma for vasopressin assay. All samples were snap frozen on dry ice then stored at −80 °C prior to analysis.

### Biomarker measurement

Vasopressin was extracted from the plasma and the concentrations were measured by radioimmunoassay (RIA) (RB-319, Eurodiagnostica AB, Malmö, Sweden), as per kit instructions with the one adjustment that an initial sample of 1 mL of plasma was reconstituted in 700 μL assay buffer following solid phase extraction. This RIA has been previously validated for use with canine plasma [20]. Plasma cortisol concentrations were measured by RIA (Coat-a-Count®, Siemens, Los Angeles, CA, USA), as per kit instructions. This RIA has previously been validated for use with canine plasma [21].

### Pharmacokinetic analysis

#### Ondansetron and metoclopramide measurement

Fifty μL sample, blank, standard or quality control (QC) were added to a 2 mL polypropylene tube. Internal standard solution (10 μL; sulpiride) and 200 μL acetonitrile (protein precipitation) were added and the tubes were vortex mixed for 5 min. Following centrifugation at 13400 xg for 2 min, the supernatant (100 μL) was diluted 1:1 with de-ionised water. Ten μL of this extract were injected onto the HPLC/MS/MS. Ondansetron and metoclopramide concentrations were measured by HPLC/MS/MS using a 10 cm × 4.6 mm SUPELCOSIL™ LC-Si HPLC Column maintained at 50 °C. This was coupled with electrospray ionisation (ESI) and tandem mass spectrometry (API 4000, Applied Biosystems). The mobile phase consisted of acetonitrile/10 mM ammonium formate (80/20 *v*/v) delivered at 1 mL/min. Positive ions were monitored in the MRM mode with m/z transitions 300 → 227 for metoclopramide, 294 → 212 for ondansetron and 342 → 112 for internal standard sulpiride. Six non-zero standards were included in each run, final concentrations of 2.5, 10, 25, 50, 75 and 100 ng/mL in plasma. Three quality controls (15, 30 and 75 ng/mL) were also analysed in duplicate in each run. A 1/x^2^ weighted linear regression was used to generate the calibration curve for both drugs.

#### Maropitant measurement

Hundred μL sample, blank, standard or quality control (QC) were added to a 96 well polypropylene plate containing 400 μL acetonitrile (protein precipitation) with 5 ng/mL of internal standard CJ-12191. The plate was sealed and centrifuged for 15 min at 1700 x *g* to pellet precipitated proteins. Ten μL of supernatant was added to a 96 well injection plate containing 790 μL water/acetonitrile with 0.1% formic acid (50/50, *v*/v). Maropitant concentrations were measured by ultra-performance liquid chromatography (UPLC) (Waters Acquity, Milford, MA, USA) using Waters ACQUITY column BEH C 1.7 μm, 2.1 × 50 mm, equipped with VanGaurd ™ Pre-Column. This was coupled with electrospray ionization (ESI) and tandem mass spectrometry (MS/MS) (API 4000, Applied biosystems/MDS Sciex, Frarmingham, MA, USA). The range of the assay is 1–1000 ng/mL. The mobile phase A consisted of 5 mM ammonium formate with 0.3% formic acid and mobile phase B consisted of acetonitrile with 0.3% formic acid. The target column was set to 55 °C. The UPLC was set to run a 1.8 min linear gradient initiated 0.2 min after sample injection from 10% to 99.9% mobile phase B for 2 mins. LC-MS/MS was set to operate in positive ion mode using the ESI source. Positive ions were monitored in the MRM mode of m/z transitions 469 → 177 for the maropitant target analyte and 455 → 163 for the internal standard CJ-12191. Eight non-zero standards were included in each run, final concentration of 1, 2, 5, 10, 50, 100, 500 and 1000 ng/mL in dog plasma. Three quality controls (3, 30 and 800 ng/mL) were also analysed in duplicate in each run. A 1/x^2^ weighted linear regression was used to generate the calibration curve.

### Statistical analysis

All statistical analyses were carried out using PASW statistics 18 v 18.0.0 (SPSS: An IBM company, Chicago, IL, USA). All data are presented as mean ± SEM and significance levels were set at *P* ≤ 0.05.

After checking normality of the distribution, correlation analyses were carried out between nausea-like behaviour and change in biomarkers, AVP and cortisol (Pearson’s coefficient). Time courses of nausea-like behaviour and change in biomarkers were analysed using a linear mixed-effect model with 1st order autoregressive covariance structure. The statistical model included the fixed effects of treatment group, time and group*time interaction, the repeated effects of time and the random effect of subject. A least significant difference *post-hoc* pairwise comparison of anti-emetic treatment was carried out at each time point to determine if there was a significant interaction between treatment and time. The area under the curve (AUC) was calculated for timecourse of change of nausea-like behaviour and biomarkers, AVP and cortisol, by determining the trapezoidal area under the curve. AUCs and the difference in the number of vomits for each antiemetic treatment group were compared using one-way ANOVA with Tukeys post hoc comparison.

Pharmacokinetic parameters and compartmental modelling was carried out using WinNonlin professional software (WinNonlin, Version 5.2, Pharsight Corp, Mountain View, CA, USA).

## Results

The repeated administration of cisplatin throughout the study was well tolerated as previously reported in Kenward et al. [16]. Each treatment group consisted of *n* = 8 except for the metoclopramide group where *n* = 7 as one dog was excluded from the last period due to the development of atopic dermatitis unrelated to the study.

### Emesis

**Fig. 2:**
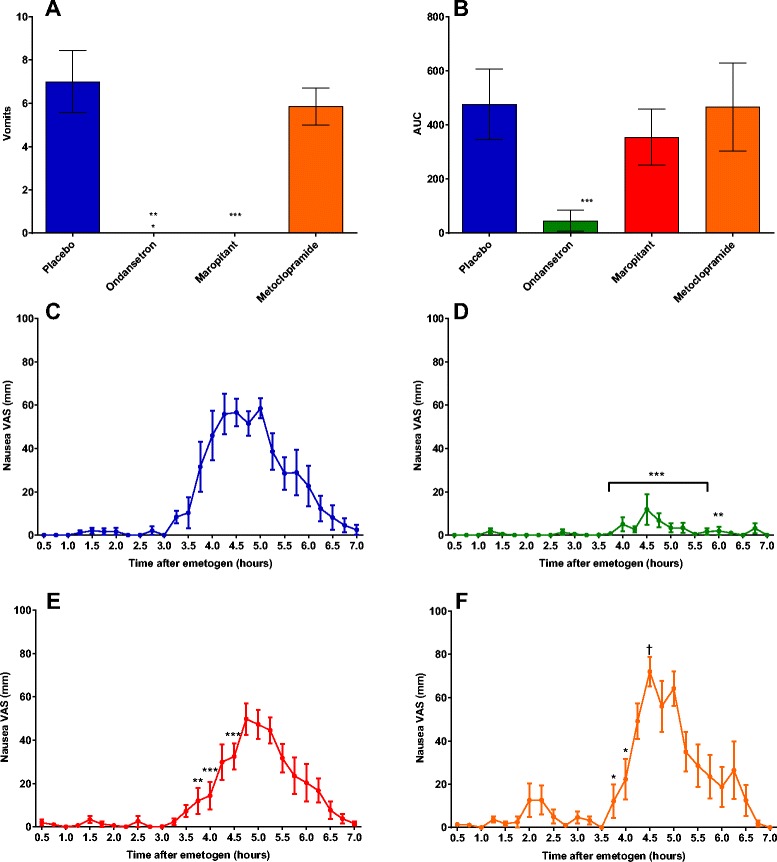
**a**-**f** Emesis and Nausea behaviour following 18 mg m^−2^ cisplatin i.v. The number of vomits (**a**) and area under the curve of nausea behaviour (**b**) for all 3 anti-emetic drugs compared to placebo. Timecourse of nausea-like behaviour (VAS) following 18 mg m^−2^ cisplatin i.v. for each treatment groups; placebo (**c**), ondansetron 0.5 mg kg^−1^ (**d**), maropitant 1 mg kg^−1^ (**e**), metoclopramide 0.5 mg kg^−1^ (**f**). Values presented as mean ± SEM, *n* = 8 per group, except metoclopramide group where *n* = 7. Significant decrease of antiemetic treated groups compared to placebo: mixed linear model (**P < 0.01, *** *P* < 0.001), Significant increase in nausea behaviour between placebo and anti-emetic treated groups: mixed linear model (†*P* < 0.05). Anti-emetic treated groups compared to placebo; Area under curve and number of vomits, ANOVA (*P < 0.05, ***P < 0.001)

The placebo treated group vomited an average number of 7 times (range 2–13 vomits; Fig. 2a). None of the dogs in either the ondansetron or maropitant treated groups vomited during the 8 h observation period. The metoclopramide treated animals vomited an average of 6 times (range of 3–10 vomits). There was no statistically significant reduction in emesis comparing the placebo and metoclopramide treated dogs.

### Signs of nausea

The onset of nausea-like behaviour in the placebo treated group occurred at t_3.5h_ and peaked at t_4.75h_ with a VAS value of 58.5 ± 4.6 mm (Fig. 2c). Nausea-like behaviour was significantly decreased in the ondansetron treated group from t_3.75 to 6h_ where the peak nausea response was VAS 11.9 ± 7.0 mm at t_4.5h_ (Fig. 2d). In both the maropitant and metoclopramide treatment groups, the onset of nausea-like behaviour was delayed. VAS scores were significantly reduced compared to placebo at t_3.75-4h_ and t_4.5_ h in the maropitant treated group and at t_3.75-4h_ in the metoclopramide treated group. In the maropitant treatment group, the peak nausea response was 49.8 ± 7.4 mm occurring at t_4.75h_ (Fig. 2e). The peak nausea response for the metoclopramide treatment group was significantly increased from placebo where the VAS score was 72 ± 6.9 mm at t_4.5h_ (Fig. 2f). The AUC for nausea-like behaviour was only significantly reduced in the ondansetron treated group compared placebo, a 90% reduction (Fig. 2b).

### Biomarkers

#### Arginine vasopressin

**Fig. 3:**
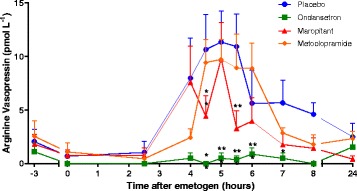
Plasma arginine vasopressin following 18 mg m^−2^ cisplatin i.v. Timecourse of change in plasma AVP following 18 mg m^−2^ cisplatin i.v. comparing placebo to each of the antiemetic treatment groups. Values presented as mean + SEM, *n* = 8 per group, except metoclopramide group where *n* = 7. Significant differences between placebo and anti-emetic treated groups: mixed linear model (**P < 0.01, *** *P* < 0.001)

In the placebo treated group the onset of AVP increase occurred at t_2.5h_ and peaked at t_5h_ with a value of 11.35 ± 2.92 pmol/L (Fig. 3). Following cisplatin administration, the AVP concentration in the ondansetron treated group did not exceed the baseline plasma AVP concentration (1.11 ± 0.84 pmol/L) at any time in the 8 h following cisplatin administration and was significantly reduced compared to placebo from t_4h_ to t_8h_. Plasma AVP was significantly decreased in the maropitant treated group compared to placebo at t_4.5h_ and t_5.5h_ but the peak of 9.79 ± 3.39 pmol/L was not significantly different from placebo. No significant difference was detected between the placebo and the metoclopramide treated group at any time point during the study. The AVP AUC of the ondansetron treated group was significantly reduced compared to placebo (*P* < 0.001). There was no significant difference in AUC between placebo and either the maropitant or the metoclopramide treated groups (Fig. 3).

#### Cortisol

**Fig. 4:**
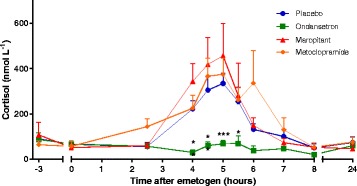
Plasma cortisol following 18 mg m^−2^ cisplatin i.v. Timecourse of change in plasma cortisol following 18 mg m^−2^ cisplatin i.v. comparing placebo to each of the antiemetic treatment groups. Values presented as mean + SEM, *n* = 8 per group, except metoclopramide group where *n* = 7. Significant differences between placebo and anti-emetic treated groups: mixed linear model (*P < 0.05, **P < 0.01, *** *P* < 0.001)

Plasma cortisol increased from baseline in the placebo treated group, the onset of increase occurred from t_2.5h,_ reaching a peak concentration of 334.05 ± 46.71 nmol/L at t_5h_ and returned to baseline by t_8h_ (Fig. 4). The ondansetron treated group had significantly lower cortisol levels compared to placebo from t_4h_ to t_5.5h_. Like the plasma AVP response, plasma cortisol of the ondansetron group did not exceed mean baseline (t_-3h_) plasma cortisol concentration of 87.21 ± 29.14 nmol/L at any time following cisplatin administration. Cortisol concentrations were not statistically different from placebo at any time point measured in either the maropitant or the metoclopramide treated groups. There was no significant difference in cortisol AUC for any of the anti-emetic drug treatment groups compared to placebo (Fig. 4).

#### Biomarkers vs nausea-like behaviour correlation

**Fig. 5:**
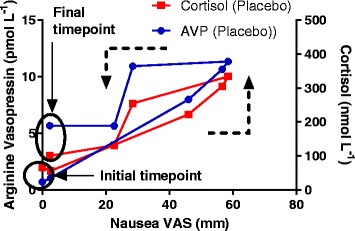
Negative Hysteresis relationship for Cortisol and arginine vasopressin (AVP) versus nausea-like behaviour (group average, *n* = 8 dogs) following 18 mg m^−2^ cisplatin i.v. The time courses of changes in both biomarkers against VAS are indicated by the dotted black arrows (anticlockwise.) AVP rises with VAS scores but persist to high level when the nausea score decreases (hysteresis loop suggesting a time delay), whereas cortisol follows nausea scores changes more closely in time

There was a weak significant correlation between AVP and VAS (*P* = 0.0065, R^2^ = 0.1362) and between Cortisol and VAS (*P* < 0.0001 and R^2^ = 0.2699). The relationship between these variables and VAS was distorted because of the time lag between the rise in VAS and the rise of the biomarkers and can be better visualised with a hysteresis plot (Fig. 5.). AVP rose with VAS scores but persisted to high level when the nausea score decreased (hysteresis loop suggesting a time delay), whereas cortisol followed nausea scores changes more closely in time.

### Pharmacokinetics

**Fig. 6:**
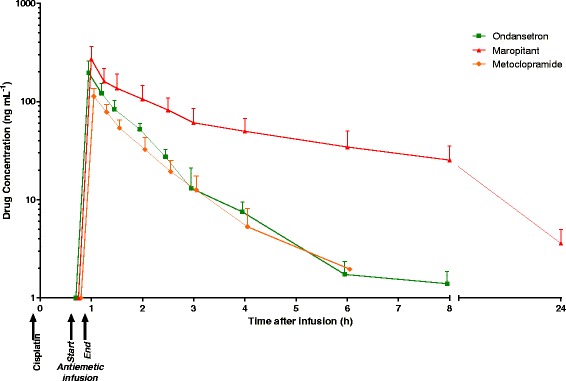
Plasma concentration-time curve for the 3 antiemetic treatments. Timecourse of change in plasma concentration of the maropirant, metoclopramide and ondansetron after intravenous injection of 1 mg kg^−1^, 0.5 mg kg^−1^ and 0.5 mg kg^−1^, respectively. Values presented as mean + SEM, *n* = 8 per group, except metoclopramide group where *n* = 7

Goodness of fit of the standard curves was *R* = 0.9986, 0.9863 and 0.9995 for ondansetron, maropitant and metoclopramide, respectively. The accuracy assays ranged between 101.3% to 108.6% for ondansetron, 82.5% to 119% for maropitant and 89.7% to 96.3% for metoclopramide,. Plasma concentration-time profiles are presented in Fig. 6. Disposition for all 3 antiemetic drugs was best described by a two-compartment model. Maximal concentration was at the completion of the 15 min intravenous infusion (t_1h_). All PK parameters are summarised in Table 1.

**Table 1:** Pharmacokinetic parameters for metoclopramide, ondansetron and maropitant after IV administration

Parameter	Units	Ondansetron (*n* = 8)	Maropitant (*n* = 8)	Metoclopramide (*n* = 7)
C_max_	μg/L	186.8 ± 60.6	247.0 ± 86.2	113.2 ± 22.4
t_1/2_	h	1.21 ± 0.51	5.62 ± 0.77	0.87 ± 0.17
AUC_0-last_	μg/L/h	176.4 ± 32.6	705.2 ± 236	111.9 ± 27.2
Cl	L/kg/h	2.90 ± 0.43	1.57 ± 0.53	4.72 ± 1.25
MRT	h	1.15 ± 0.43	6.68 ± 1.30	1.04 ± 0.16

Legend: All results are presented as the arithmetic mean ± SD except t_1/2_ which is presents as the harmonic mean ± Pseudo SD

Ondansetron concentrations in plasma were below the lower limit of quantification in 3 of 8 dogs at t_7h_ and in all animals by t_24h_. Maropitant was still detectable in the plasma of all dogs at t_24h_. Metoclopramide concentrations in plasma fell below the lower limit of quantification in 3 of 7 dogs at t_5h_ and in all animals at t_7h_.

## Discussion

The present study, using a low dose model of cisplatin to induce nausea and vomiting, has demonstrated differential relative anti-emetic and anti-nausea efficacy within three classes of anti-emetic drugs when used at dose rates previously shown to inhibit emesis in the dog. Ondansetron inhibited both emesis and all behavioural signs of nausea completely. Maropitant inhibited emesis completely but only partially reduced the behavioural signs of nausea at the early time points. Metoclopramide had no significant effects on the number of episodes of vomiting and very little effect on the behavioural signs of nausea. These results support the hypothesis that emesis is easier to prevent than nausea by reducing the neuronal signals to below the threshold required to trigger emesis. By contrast, nausea, being a graded phenomenon, persisted in the presence of concentrations of maropitant which prevented dogs vomiting. The objective biomarkers of nausea, plasma AVP and cortisol identified by our previous characterisation of this low dose cisplatin model [16] add weight to the conclusions made based on subjective behavioural observations of nausea.

Cisplatin-induced nausea and vomiting has a biphasic time-course in human subjects. The ‘acute’ phase of nausea and vomiting occurs in the first 24 h following treatment. Nausea and vomiting occurring more than 24 h following treatment is referred to as the ‘delayed phase’ and can persist for 3–5 days [22, 23]. The present study focused on the initial ‘acute’ phase of cisplatin-induced nausea and vomiting which is thought to be predominantly peripherally mediated. Cisplatin causes the release of 5-HT from the gut which stimulates abdominal vagal afferent neurons that project to the ‘vomiting centre’ in the brainstem [22]. Cisplatin emesis can be abolished in dogs by abdominal vagotomy or the systemic administration of 5-HT_3_ antagonists but not by the central administration of 5-HT_3_ antagonists into the cerebral ventricles [24]. The ‘delayed’ phase is mediated mainly by a central mechanism of action. The area postrema (commonly referred to as the chemoreceptor trigger zone) located in the brainstem is thought to be stimulated by cisplatin, its metabolites, or gut peptides released in response to the effects of cisplatin on the intestine [22]. Ablation of the area postrema in ferrets abolished the delayed emetic response to cisplatin but bilateral vagotomy did not [23].

Nausea, unlike emesis, is a multi-dimensional experience incorporating emotional and affective components in addition to the physiological response. The sensation of nausea is thought to arise from activation of cortical structures involved in conscious perception [9, 25, 26]. The mechanisms by which cisplatin induces nausea are less clear. If nausea was solely induced by stimulation of the emetic pathways then it would be expected that abolition of emesis would also abolish nausea, however, this is not the case [18]. The results of the present study show, in the maropitant treated group, that nausea-like behaviour can be detected in the absence of an emetic response.

The low dose cisplatin model used in the present study has greater utility for the investigation of nausea in the dog without the large number of emetic events induced by a clinical dose of cisplatin. A high number of emetic events may bias the observer when judging the severity of nausea as a function of the emesis observed rather than as a distinct sensation with specific associated behaviours, which may be obscured by frequent vomiting and retching. The behavioural assessment in the present study judged the maximum VAS score as the ‘worst possible nausea’ for the low dose model of cisplatin gained from previous experience with the model [16]. This adjustment of the behavioural scale means it is possible for a maximum VAS score to be given and allows for the greatest differentiation of the anti-nausea effects of the anti-emetic drugs tested in the model. Careful habituation of the dogs to the context of the experiment was necessary in order for the main investigator to learn what their normal individual behaviour was within that situation in the absence of cisplatin treatment.

AVP and cortisol have been previously found to be correlates of the behavioural signs of nausea induced by cisplatin [16] but it was not clear if the emetic response also contributed to the change in these biomarkers. The results of the present study support AVP and cortisol as specific biomarkers of nausea rather than emesis. Complete inhibition of the behavioural signs of nausea in the ondansetron treated group results in reduction of AVP and cortisol to baseline levels whereas complete inhibition of emesis and only partial inhibition of the behavioural aspects of the nausea in the maropitant treated group led to only partial inhibition of AVP and no inhibition of cortisol. AVP appears to provide the more sensitive marker of nausea-like behaviour as partial inhibition of nausea-like behaviour by maropitant was identified through a significantly reduced AVP compared to placebo at t_4.5h_, which was not evident in the cortisol response. The relationship between these nausea biomarkers and the nausea-like behavioural response requires further characterisation in a study using a range of doses of ondansetron and maropitant that describe the full concentration anti-nausea effect relationship of these two drugs more completely than is possible from the data generated by the present study.

D_2_ receptor antagonists, such as metoclopramide, have both central and peripheral mechanisms. They act centrally on D_2_ receptors in emetic brainstem regions, such as the area postrema and dorsal vagal complex [27]. Peripherally, they are prokinetic, resolving gastric dysrhythmias which are associated with nausea and emesis [18]. Metoclopramide also has a weak 5-HT_3_ antagonist action providing some of its anti-emetic efficacy [28]. Metoclopramide has previously demonstrated some anti-nausea efficacy in humans receiving cisplatin treatment. Allan et al. [29] found that metoclopramide abolished or achieved major control in 26% and 46% of cisplatin treated patients respectively. Metoclopramide also completely controlled nausea in 24% of patients and achieved major control of nausea in 47% of patients; this was increased to 32% and 62% respectively when combined with dexamethasone [29]. Metoclopramide was also found to significantly reduce the duration of nausea induced by cisplatin chemotherapy [3]. Metoclopramide is used frequently in veterinary medicine to control nausea and vomiting. A dose of 1–3 mg/kg of metoclopramide administered by subcutaneous injection has been found to reduce cisplatin-induced emesis in the dog [30]. However, in the current study metoclopramide did not exhibit any anti-emetic or anti-nausea efficacy and nausea-like behaviours actually had a higher peak compared to placebo. One explanation for this observation could be the extra-pyramidal side effects of the drug increasing restlessness which increased the nausea-like behavior score above that of placebo. Metoclopramide was cleared the fastest of the all three antiemetic drugs. It is possible a higher doses of metoclopramide may have produced a greater anti-emetic and anti-nausea efficacy, however the administered dose of 0.5 mg/kg was at the highest end of the recommended dose for dogs which is 0.25–0.5 mg/kg every 6–8 h [31] and worsening extra-pyamidal side effects are seen in dogs given high doses of metoclopramide [32].

Ondansetron was the first-in-class 5-HT_3_ antagonist acting via both central and peripheral mechanisms. Peripherally, 5-HT_3_ antagonists block the activation of abdominal vagal afferents by emetic stimuli in the gastrointestinal tract [33]. Centrally, blockade of 5-HT_3_ receptors on the terminals of vagal inputs into emetic brain stem regions prevents emetic stimuli from reaching the vomiting centre [28]. Ondansetron significantly reduced the number of episodes of emesis, increased the latency to emesis and decreased nausea VAS scores compared to placebo in patients receiving cisplatin chemotherapy [4]. The level of control of nausea and emesis was significantly greater with ondansetron compared to metoclopramide and ondansetron treatment was preferred by patients [34, 35]. Ondansetron provided greatest efficacy in the control of acute nausea following cisplatin treatment, whereas metoclopramide was found to be significantly more efficacious at controlling delayed nausea [34]. The combination of ondansetron with dexamethasone further improved the level of control of nausea [36, 37]. Ondansetron also exhibits anti-emetic and anti-nausea efficacy in animal models of nausea and vomiting. Ondansetron significantly inhibits emesis induced by cisplatin in ferrets [38] and by methotrexate in dogs [39]. In a lycorine model of nausea and vomiting in beagle dogs, ondansetron significantly reduced emesis and also exhibited significant anti-nausea activity [40].

NK_1_ antagonists are the most recent class of antiemetic drugs, aprepitant being approved by the FDA in 2003 for use in humans and maropitant being approved in 2007 for use in the dog. The anti-emetic properties are thought to occur as a result of the blockade of NK_1_ receptors in emetic brainstem regions, including the area postrema and the nucleus tractus solitarius [41, 42]. The addition of aprepitant to standard antiemetic therapy with dexamethasone and a 5-HT_3_ antagonist improved the control of chemotherapy-induced nausea and vomiting especially in the delayed phase. Aprepitant significantly reduced emesis overall and in both the acute and delayed phases [43–45]. Nausea was significantly reduced overall and in the delayed phase but not the acute phase [43, 45]. In a clinical dose (70 mg/m^2^) cisplatin model of nausea and vomiting in the dog, maropitant significantly reduced emesis and had anti-nausea efficacy suppressing nausea-like behaviours across the full time-course and significantly decreasing the peak nausea response [15]. In the present study, maropitant exhibited less anti-nausea efficacy than reported by de la Puente Redondo et al. [15]. It is possible that maropitant may have greater efficacy against the more severe nausea induced by the higher dose of cisplatin. For this explanation to be correct would require severe nausea to be induced by different mechanisms at higher doses of cisplatin which seems unlikely. An alternative explanation is that the significant reduction of emesis induced by maropitant treatment may have caused a bias towards reduced nausea scores in the high dose cisplatin study, a bias that is not so much of a problem in the low dose cisplatin model used in the present study. Another difference between the present study and that of de la Puente Redondo et al. [15] is the route of administration of maropitant. In the present study maropitant was administered by i.v. infusion whereas it was given subcutaneously in the study by de la Puente Redondo et al. [15]. The alternative route of administration used in the present study leads to a higher initial concentration of maropitant and a shorter half-life compared to s.c. administration. Maropitant plasma concentrations could have been at sub-therapeutic concentrations during the nausea response in the present study. However, this is improbable as an antiemetic and anti-nausea effect demonstrated 19 h following administration 1 mg/kg maropitant s.c. [15] when plasma concentrations would certainly be lower than those during the nausea response in the present study.

The results of the present study suggest that both 5-HT_3_ and NK_1_ receptors are an integral part of the emetic pathway activated by cisplatin which results in activation of the emetic reflex. The sensation of nausea is produced in cortical forebrain regions [46], increased activity was recorded in the left amygdala, the ventral putamen and the putative locus coeruleus [47]. Nausiogenic signals travel from the vomiting centre via rostrally projecting pathways to the forebrain and nuclei controlling the physiological response to nausea sensation (e.g. salivating, restlessness). The ability of ondansetron to reduce cisplatin-induced nausea would suggest that 5-HT_3_ receptors have a role in transmitting nausea stimuli, either from the brainstem, the periphery or both, whereas NK_1_ receptors are limited to a central emetic triggering mechanism.

## Conclusions

In a low dose cisplatin model of nausea and emesis in the dog, NK_1_ antagonist demonstrated good anti-emetic activity but limited anti-nausea effect. The 5-HT_3_receptor antagonist ondansetron was most effective at treating both cisplatin-induced nausea and vomiting with associated reductions in nausea biomarkers AVP and cortisol. Further study using escalating doses of anti-emetics as interventions to inhibit nausea induced by different stimuli would be beneficial to determine the full PK/PD relationship of the anti-nausea effects of anti-emetics and explore the utility of nausea biomarkers in studying the pathways that can give rise to nausea.

## References

[1] Stern RM, Koch KL, Andrews PLR (2011). Nausea: mechanisms and management.

[2] Kenward H, Pelligand L, Savary-Bataille K, Elliott J (2015). Nausea: current knowledge of mechanisms, measurement and clinical impact. Vet J.

[3] Gralla RJ, Itri LM, Pisko SE, Squillante AE, Kelsen DP, Braun DW, Bordin LA, Braun TJ, Young CW (1981). Antiemetic efficacy of high-dose metoclopramide: randomized trials with placebo and prochlorperazine in patients with chemotherapy-induced nausea and vomiting. N Engl J Med.

[4] Cubeddu LX, Hoffmann IS, Fuenmayor NT, Finn AL (1990). Efficacy of ondansetron (GR 38032F) and the role of serotonin in cisplatin-induced nausea and vomiting. N Engl J Med.

[5] Poli-Bigelli S, Rodrigues-Pereira J, Carides AD, Julie Ma G, Eldridge K, Hipple A, Evans JK, Horgan KJ, Lawson F (2003). Addition of the neurokinin 1 receptor antagonist aprepitant to standard antiemetic therapy improves control of chemotherapy-induced nausea and vomiting. Results from a randomized, double-blind, placebo-controlled trial in Latin America. Cancer.

[6] Pirri C, Bayliss E, Trotter J, Olver IN, Katris P, Drummond P, Bennett R (2013). Nausea still the poor relation in antiemetic therapy? The impact on cancer patients' quality of life and psychological adjustment of nausea, vomiting and appetite loss, individually and concurrently as part of a symptom cluster. Support Care Cancer.

[7] Morrow GR, Roscoe JA, Hickok JT, Andrews PR, Matteson S (2002). Nausea and emesis: evidence for a biobehavioral perspective. Support Care Cancer.

[8] Holmes AM, Rudd JA, Tattersall FD, Aziz Q, Andrews PL (2009). Opportunities for the replacement of animals in the study of nausea and vomiting. Br J Pharmacol.

[9] Horn CC (2008). Why is the neurobiology of nausea and vomiting so important?. Appetite.

[10] Sanger GJ, Andrews PL (2006). Treatment of nausea and vomiting: gaps in our knowledge. Auton Neurosci: basic & clinical.

[11] Zamble DB, Lippard SJ (1995). Cisplatin and DNA repair in cancer chemotherapy. Trends Biochem Sci.

[12] Roila F, Herrstedt J, Aapro M, Gralla RJ, Einhorn LH, Ballatori E, Bria E, Clark-Snow RA, Espersen BT, Feyer P (2010). Guideline update for MASCC and ESMO in the prevention of chemotherapy- and radiotherapy-induced nausea and vomiting: results of the Perugia consensus conference. Ann Oncol.

[13] Mutoh M, Imanishi H, Torii Y, Tamura M, Saito H, Matsuki N (1992). Cisplatin-induced emesis in Suncus Murinus. Jpn J Pharmacol.

[14] Percie du Sert N, Rudd J, Apfel C, Andrews P (2011). Cisplatin-induced emesis: systematic review and meta-analysis of the ferret model and the effects of 5-HT3 receptor antagonists. Cancer Chemother Pharmacol.

[15] de la Puente-Redondo VA, Tilt N, Rowan TG, Clemence RG (2007). Efficacy of maropitant for treatment and prevention of emesis caused by intravenous infusion of cisplatin in dogs. Am J Vet Res.

[16] Kenward H, Pelligand L, Elliott J (2014). Assessment of low-dose cisplatin as a model of nausea and emesis in beagle dogs, potential for repeated administration. Exp Brain Res.

[17] Otto B, Riepl RL, Klosterhalfen S, Enck P (2006). Endocrine correlates of acute nausea and vomiting. Auton Neurosci..

[18] Stern RM, Koch KL, Andrews P (2011). Nausea: mechanisms and management.

[19] Henry CJ, Higginbotham ML (2009). Cancer Management in Small Animal Practice, first edition edn.

[20] Tidholm A, Haggstrom J, Hansson K (2005). Vasopressin, cortisol, and catecholamine concentrations in dogs with dilated cardiomyopathy. Am J Vet Res.

[21] Olsson K, Bergstrom A, Kindahl H, Lagerstedt AS (2003). Increased plasma concentrations of vasopressin, oxytocin, cortisol and the prostaglandin F2alpha metabolite during labour in the dog. Acta Physiol Scand.

[22] Hesketh PJ (2008). Chemotherapy-induced nausea and vomiting. N Engl J Med.

[23] Percie du Sert N, Rudd JA, Moss R, Andrews PL (2009). The delayed phase of cisplatin-induced emesis is mediated by the area postrema and not the abdominal visceral innervation in the ferret. Neurosci Lett.

[24] Fukui H, Yamamoto M, Sato S (1992). Vagal afferent fibers and peripheral 5-HT3 receptors mediate cisplatin-induced emesis in dogs. Jpn J Pharmacol.

[25] Mulak A, Kahane P, Hoffmann D, Minotti L, Bonaz B. Brain mapping of digestive sensations elicited by cortical electrical stimulations. Neurogastroenterol & Motility. 2008;20(6):588–96. 10.1111/j.1365-2982.2007.01066.x 18208482

[26] Sanger GJ, Andrews PL (2006). Treatment of nausea and vomiting: gaps in our knowledge. Auton Neurosci.

[27] Hyde TM, Knable MB, Murray AM (1996). Distribution of dopamine D1-D4 receptor subtypes in human dorsal vagal complex. Synapse.

[28] Freeman A, Cunningham K, Tyers M (1992). Review paper: selectivity of 5-HT3 receptor antagonists and anti-emetic mechanisms of action. Anti-Cancer Drugs.

[29] Allan SG, Cornbleet MA, Warrington PS, Golland IM, Leonard RC, Smyth JN (1984). Dexamethasone and high dose metoclopramide: efficacy in controlling cisplatin induced nausea and vomiting. Br Med J (Clin Res Ed).

[30] Gylys JA, Doran KM, Buyniski JP (1979). Antagonism of cisplatin induced emesis in the dog. Res Commun Chem Pathol Pharmacol.

[31] Ramsey I., (Ed.). Small Animal Formulary [8th Edition]. BSAVA Publications: Gloucester; 2014.

[32] Dowling PM. Prokinetic drugs: metoclopramide and cisapride. The Canadian veterinary journal = La revue veterinaire canadienne 1995;36(2):115–116. PMC1686860 7728729

[33] Rojas C, Slusher BS (2012). Pharmacological mechanisms of 5-HT(3) and tachykinin NK(1) receptor antagonism to prevent chemotherapy-induced nausea and vomiting. Eur J Pharmacol.

[34] De Mulder PH, Seynaeve C, Vermorken JB, van Liessum PA, Mols-Jevdevic S, Allman EL, Beranek P, Verweij J (1990). Ondansetron compared with high-dose metoclopramide in prophylaxis of acute and delayed cisplatin-induced nausea and vomiting. A multicenter, randomized, double-blind, crossover study. Ann Intern Med.

[35] Marty M, Pouillart P, Scholl S, Droz JP, Azab M, Brion N, Pujade-Lauraine E, Paule B, Paes D, Bons J (1990). Comparison of the 5-hydroxytryptamine3 (serotonin) antagonist ondansetron (GR 38032F) with high-dose metoclopramide in the control of cisplatin-induced emesis. N Engl J Med.

[36] Roila F, Tonato M, Cognetti F, Cortesi E, Favalli G, Marangolo M, Amadori D, Bella MA, Gramazio V, Donati D (1991). Prevention of cisplatin-induced emesis: a double-blind multicenter randomized crossover study comparing ondansetron and ondansetron plus dexamethasone. J Clin Oncol..

[37] Smith DB, Newlands ES, Rustin GJ, Begent RH, Howells N, McQuade B, Bagshawe KD (1991). Comparison of ondansetron and ondansetron plus dexamethasone as antiemetic prophylaxis during cisplatin-containing chemotherapy. Lancet (London, England).

[38] Rudd JA, Naylor RJ (1994). Effects of 5-HT3 receptor antagonists on models of acute and delayed emesis induced by cisplatin in the ferret. Neuropharmacology.

[39] Fukui H, Yamamoto M (1999). Methotrexate produces delayed emesis in dogs: a potential model of delayed emesis induced by chemotherapy. Eur J Pharmacol.

[40] Kretzing S, Abraham G, Seiwert B, Ungemach FR, Krugel U, Teichert J, Regenthal R (2011). In vivo assessment of antiemetic drugs and mechanism of lycorine-induced nausea and emesis. Arch Toxicol.

[41] Andrews PL, Kovacs M, Watson JW (2001). The anti-emetic action of the neurokinin(1) receptor antagonist CP-99,994 does not require the presence of the area postrema in the dog. Neurosci Lett.

[42] Tattersall FD, Rycroft W, Francis B, Pearce D, Merchant K, MacLeod AM, Ladduwahetty T, Keown L, Swain C, Baker R (1996). Tachykinin NK1 receptor antagonists act centrally to inhibit emesis induced by the chemotherapeutic agent cisplatin in ferrets. Neuropharmacology.

[43] Chawla SP, Grunberg SM, Gralla RJ, Hesketh PJ, Rittenberg C, Elmer ME, Schmidt C, Taylor A, Carides AD, Evans JK (2003). Establishing the dose of the oral NK1 antagonist aprepitant for the prevention of chemotherapy-induced nausea and vomiting. Cancer.

[44] Hesketh PJ, Grunberg SM, Gralla RJ, Warr DG, Roila F, de Wit R, Chawla SP, Carides AD, Ianus J, Elmer ME (2003). The oral neurokinin-1 antagonist aprepitant for the prevention of chemotherapy-induced nausea and vomiting: a multinational, randomized, double-blind, placebo-controlled trial in patients receiving high-dose cisplatin--the Aprepitant protocol 052 study group. J Clin Oncol..

[45] Warr DG, Grunberg SM, Gralla RJ, Hesketh PJ, Roila F, Wit R, Carides AD, Taylor A, Evans JK, Horgan KJ (2005). The oral NK(1) antagonist aprepitant for the prevention of acute and delayed chemotherapy-induced nausea and vomiting: Pooled data from 2 randomised, double-blind, placebo controlled trials. European journal of cancer (Oxford, England : 1990).

[46] Horn CC, Ciucci M, Chaudhury A (2007). Brain Fos expression during 48 h after cisplatin treatment: neural pathways for acute and delayed visceral sickness. Auton Neurosci.

[47] Napadow V, Sheehan JD, Kim J, Lacount LT, Park K, Kaptchuk TJ, Rosen BR, Kuo B (2013). The brain circuitry underlying the temporal evolution of nausea in humans. Cereb Cortex.

